# Establishment, validation and application of a spectrophotometric method for the accurate determination of carbonic anhydrase activity

**DOI:** 10.1039/d5ra06480e

**Published:** 2025-12-01

**Authors:** Xiaoxiao Liu, Renci Tian, Haoran Mi, Wuxia Guo, Gang Chen

**Affiliations:** a School of Bioengineering, Zhuhai Campus of Zunyi Medical University 519041 Zhuhai City Guangdong Province China biomedicinechen@163.com

## Abstract

Carbonic anhydrase (CA) has been proven to be a rather important enzyme in both medical research and industrial development, leading to great application value in accurately quantifying CA enzymatic activity. This study focuses on developing a novel spectrophotometric method for measuring the CA enzyme activity with enhanced precision, reliability, and an ultra-low limit of detection (LOD) through the transition of changing pH values into a stable UV absorbance signal by the colorimetric probe of bromothymol blue (BTB), thereby addressing the limitations of existing assay techniques. Through the establishment of a colorimetric reaction system, optimization of the CA assay reaction system, and data analysis by both least squares and two-point methods, CA conventional and trace standard curves in the range of 2–2000 U mL^−1^ were obtained under the following conditions: a wavelength of 616 nm, a BTB concentration of 0.015 g L^−1^, a temperature of 20 °C, and a Tris–HCl concentration of 0.028 mol L^−1^. In addition, the results from the methodology validation revealed an LOD value of 2 U mL^−1^, excellent repeatability, precision and recoveries within the investigated range. Finally, the application exploration, which included CA activity testing in human blood samples and pharmacokinetic research in rats, exhibited precise and stable results for clinical testing and enhanced linearity fitting of the pharmacokinetic curve, with increased resistance to interferences from hemoglobin (Hb) and other plasma proteins. The established spectrophotometric method provides a novel reliable analytical approach for CA activity measurement, pharmaceutical research and even future application prospects in environmental protection and other industrial fields.

## Abstract 2

Detection of carbonic anhydrase activity using bromothymol blue and an ultraviolet spectrophotometer.

## Graphical abstract

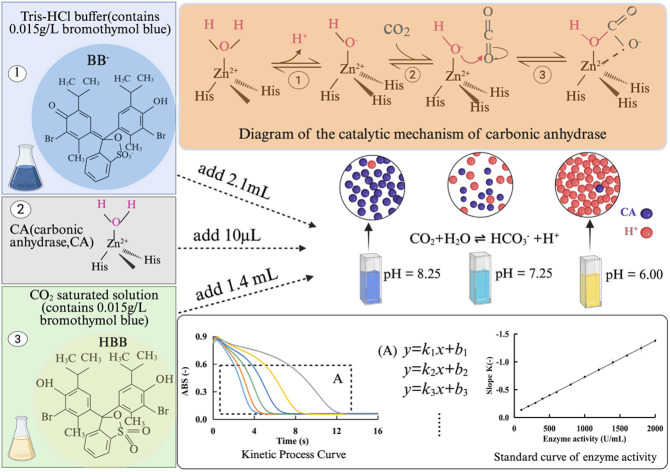

## 1 Introduction

Since its discovery by Meldrum and Roughton in 1933, carbonic anhydrase (CA) has gained increasing attention due to its broad distribution in biological systems and critical role in physiological processes.^1–3^ As an enzyme family with eight members,^4,5^ CA catalyses CO_2_ hydration to bicarbonate: it first deprotonates water to generate hydroxide ions, which then react nucleophilically with CO_2_, reversibly accelerating the reaction.^6–10^

With the further understanding of CA operation mechanisms under normal physiological processes,^11–13^ a comprehensive understanding of CA's catalytic mechanism will support the development of CA-targeted medications.^14,15^ In the field of medicine, CA plays crucial roles in numerous vital bodily physiological functions^16,17^ and regulates key physiological functions, including facilitating CO_2_ transport in erythrocytes to indirectly modulate hemoglobin oxygen-carrying capacity,^7,18^ maintaining gastric epithelium pH by neutralizing protons,^19^ sustaining renal acid–base balance *via* bicarbonate reabsorption and other functions.^20,21^ Furthermore, a series of studies have confirmed CA's importance in disease treatment, as it is directly linked to the onset of physiological abnormalities and the progression of pathological processes.^22,23^ Research on certain CA isozymes, like CA IX and CA XII,^24–26^ has led to innovative concepts for cancer diagnostics and treatment because they are important in tumor cell proliferation, invasion, and metastasis.^27–30^ Additionally, CA inhibitors have shown significant potential in the management or prevention of epilepsy,^31^ obesity,^32^ osteoporosis,^33^ infectious diseases (*e.g.*, antifungal,^34^ inhibition of antimicrobial resistance,^2,35^ the treatment of malaria,^36^ and chronic mountain sickness alleviation).^37^ Research on CA activators has also introduced several novel approaches, particularly in the treatment of Alzheimer's disease, thereby opening promising new avenues for scientific exploration.^38,39^

The CA activity assay method was first researched by Meldrum and Roughton in 1933.^1^ The developed technology was bottlenecked by enzyme inactivation, diffusion artifacts and interference from phosphate buffer. Although the measurement stability was improved in later boat manometer approaches, the complex operations and error arising during the pH-induced changes along with the vibrations in the detection readings consistently limited their reliability, thus leading to poor reliability and low precision.^40^ Therefore, colorimetric/potentiometric techniques were adopted by Wilbur and Anderson for CA activity detection.^41^ In 2022, using UV/Vis spectrophotometric methods with phenol red as the color indicator, Kim and Jo further enhanced the reliability of the CA activity detection, but retained a high limit of detection (LOD), incompatibility with immobilized enzymes, and absorbance interference.^42^ Thus, the development of a CA activity measurement method with high accuracy and stable reliability still remains a research topic of great significance.

**Fig. 1:**
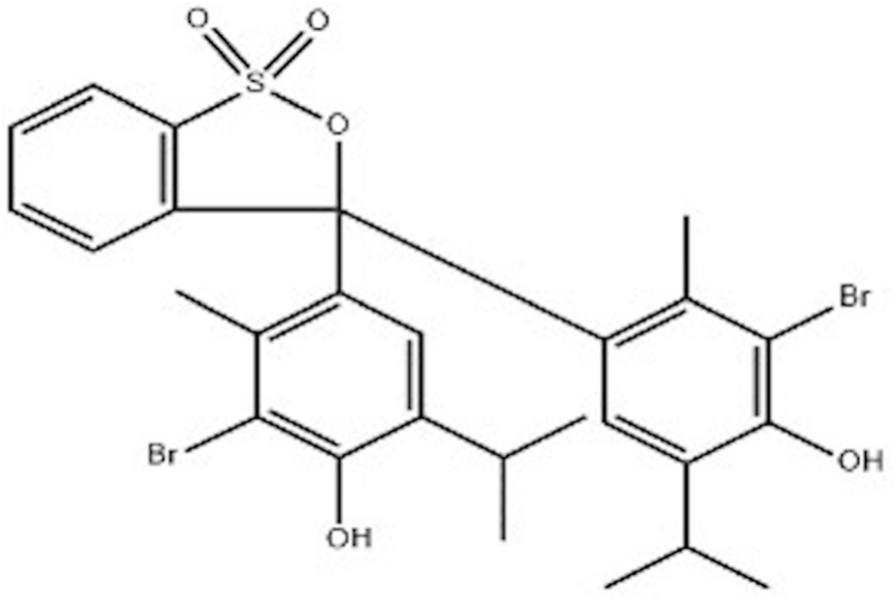
The chemical structure of BTB.

BTB is an acid–base indicator with the characteristic of presenting a yellow color under acidic conditions and blue color under alkaline conditions, which stem from the protonation or deprotonation reaction of its phenolic hydroxyl (–OH) in solutions with different pH values, leading to changes in the molecular structure and subsequent color changes (Fig. 1).^43,44^ This color change arises from the protonation/deprotonation of its phenolic hydroxyl groups, altering the molecular structure: protonation in acidic solutions (high H^+^) yields yellow BTB, while deprotonation with increasing pH (low H^+^) shifts the color to green and then blue,^45^ making BTB widely useful for acid–base detection.^46,47^

In this study, a spectrophotometric technique based on the BTB indicator was developed for CA activity determination, converting pH changes during CO_2_ hydration into stable electrical signals for rapid, reliable assessment. We established the colorimetric system, optimized the reaction conditions, and generated the standard curves. Validation included reproducibility, precision, and recovery; LOD was determined for future clinical/pharmaceutical use. Preliminary applications included CA detection in human blood and CA activity pharmacokinetic monitoring in rat plasma. With its simplicity, low LOD, and high precision, this assay provides a robust tool for CA-related research and drug development.

## 2 Materials and methods

### 2.1 Materials

All experiments were performed in accordance with the guidelines of measures for the Ethical Review of Biomedical Research Involving Human Subjects, and experiments were approved by the ethics committee at Zunyi Medical University. Informed consents were obtained from the human participants of this study.

All animal procedures were performed in accordance with the guidelines for the Care and Use of Laboratory Animals of Zunyi Medical University, and experiments were approved by the Animal Ethics Committee of Zunyi Medical University.

NaCl (AR. Sinopharm Chemical Reagent Co., Ltd), Tris-base (AR. Saiguo Biological Technology Co., Ltd), Tris–HCl (AR. Saiguo Biological Technology Co., Ltd), KH_2_PO_4_ (AR. Tianjin Damao Chemical Reagent Co., Ltd), K_2_HPO_4_ (AR. Tianjin Damao Chemical Reagent Co., Ltd), bromothymol blue (AR. Tianjin Fine Chemical Technology Development Center), isoflurane (RWD Life Science Co., Ltd), low molecular weight heparin sodium (Shanghai McLean Biopharmaceutical Co., Ltd), CA (AR. Sigma-Aldrich Co., Ltd), NaOH (AR. Sinopharm Chemical Reagent Co., Ltd), and other reagents used in this study were purchased in analytical grade.

### 2.2 Instrumentation

Ultraviolet-visible spectrophotometer (GE evolution 350, Thermo Scientific), low-speed refrigerated centrifuge (L4-4KR, Hunan Kecheng Instrument Equipment Co., Ltd, China), high-speed refrigerated centrifuge (L4-4KR, Hunan Kecheng Instrument Equipment Co., Ltd, China), conductivity meter (DDSJ-308A, Shanghai Inesa Scientific Instrument Co., Ltd), chromatography column (NmXK5040-AF, Suzhou Nano Micro Technology Co., Ltd), electronic balance (XPR 105 DR, Mettler Toledo International Limited), collecting type constant temperature heating magnetic stirrer (DF-101S, Yuhua), ultra-pure water system (CM-RO-C2, Ningbo Jinbangda Intelligent Sales Equipment Co., Ltd, China), pH meter (FiveEasy, Mettler Toledo, Switzerland), electronic balance (FA2104, Hangzhou Wante Weighing Apparatus Co., Ltd).

### 2.3 Establishment of the colorimetric reaction system

#### 2.3.1 Measurement wavelength investigation

A series of mixture solutions containing 0.015 g per L BTB and 0.02 mol per L Tris–HCl buffer with pH values of 6.25, 6.75, 7.25, 7.75 and 8.25 were prepared and then analysed by UV-spectrophotometric wavelength scanning with 0.02 mol per L Tris–HCl solution as the blank. The obtained UV-spectral curves with the wavelength range of 200–850 nm were utilized to investigate the influence of the pH values on the UV-spectral characteristics of the BTB solution. The absorbance difference between these UV-spectral curves were systematically examined to confirm the suitable wavelength for the CA activity measurement.

#### 2.3.2 Correlation between the concentration and absorbance of the BTB solution

Based on a series of 0.02 mol per L Tris–HCl buffer solutions prepared across a range of pH values (8.25, 7.75, 7.25, 6.75, 6.25), the concentrations of BTB in the Tris–HCl buffer solutions at each pH value were set as 0.005 g L^−1^, 0.010 g L^−1^, 0.015 g L^−1^, 0.020 g L^−1^, 0.035 g L^−1^, and 0.050 g L^−1^, respectively. To quantify the correlation between the concentration and absorbance characteristic value, the prepared BTB solutions with the concentration gradient at each experimental pH value were UV-spectrophotometrically measured at 616 nm, with the Tris–HCl blank buffer serving as the reference. Each sample was measured in triplicate and the average values were used for the linear analysis with concentration *vs. A*_616_ values.

#### 2.3.3 Correlation between pH value and absorbance of BTB solution

A series of 0.02 mol per L Tris–HCl buffer solutions were prepared at different gradient BTB concentrations (0.005 g L^−1^, 0.010 g L^−1^, 0.015 g L^−1^, 0.020 g L^−1^, 0.035 g L^−1^, and 0.050 g L^−1^), with the pH values of the above solution at each BTB concentration set at 8.25, 7.75, 7.25, 6.75 and 6.25, respectively. The UV-spectrophotometer was used to measure the absorbance value of the experimental samples at 616 nm, with the Tris–HCl blank buffer as the reference. By taking the average value of three measurements for each sample, the final *A*_616_ values were determined for the linear analysis with pH values *vs. A*_616_.

### 2.4 Optimization of the reaction system for the CA enzyme activity measurement

#### 2.4.1 Enzymatic reaction curves for the CA enzyme activity measurement

A spectrophotometer was used for the preparation of the enzymatic reaction curves for the CA enzyme activity measurement. The CO_2_ saturated solution and Tris–HCl buffer solution with 0.015 g per L BTB were prepared before use, and then the CA sample solution (concentration range from 0–2000 U mL^−1^) was prepared through dilution of the stock solution using 0.05 mol L^−1^ phosphate buffer. Before the measurement, the UV-spectrophotometer was set at the wavelength of 616 nm, temperature of 20 °C and kinetic selection mode, and then the detection could be immediately started following the addition of 10 µL CA enzyme solution, 2.1 mL Tris–HCl buffer and 1.4 mL CO_2_-saturated solution into the cuvette. The absorbance values of the mixture solution at 616 nm were recorded as the CO_2_ hydration proceeded, and the enzymatic reaction curve (*A*_616_*vs.* time) was obtained after the reaction process.

#### 2.4.2 Assessment of BTB effect on the detection of CA enzyme activity

As the colorimetric probe, the effects of BTB on the CA enzyme activity measurement were evaluated by measuring the CA enzymatic reaction time during the CA activity detection process. A series of concentrations of CA enzyme activities were prepared at 100 U mL^−1^, 1000 U mL^−1^, and 1800 U mL^−1^ in the sample solution, in which the BTB concentrations were set to 0.005 g L^−1^, 0.010 g L^−1^, and 0.015 g L^−1^ with each CA activity concentration. The CA enzymatic reaction curves with different BTB concentrations were then measured according to Section 2.3.4. Then, the reaction time was recorded and the influence of BTB on the CA enzyme activity measurement was subsequently evaluated through the comparison of the enzymatic reaction periodic time.

#### 2.4.3 Effect of the Tris–HCl buffer concentration on the CA enzyme activity detection

During the CA enzymatic reaction, the Tris–HCl buffer concentration in the detection system remarkably influenced the reaction kinetic curve and the periodic time. Thus, the Tris–HCl buffer concentration was used for the CA enzyme activity measurement. In this study, the investigated Tris–HCl buffer concentrations were set at a gradient of 0.015 mol L^−1^, 0.020 mol L^−1^, 0.025 mol L^−1^, 0.028 mol L^−1^, 0.030 mol L^−1^, 0.035 mol L^−1^, and 0.040 mol L^−1^. The CA enzyme activities for the reaction kinetic curves were set in the value range of 100–2000 U mL^−1^. Then, the enzymatic reaction kinetic curves were obtained as described in Section 2.3.4, and the impact of the Tris–HCl buffer concentration on the CA enzyme activity measurement was evaluated.

#### 2.4.4 Effect of temperatures on the CA enzyme activity detection

By setting a sequence of temperature gradients of 0 °C, 15 °C, 20 °C, 25 °C, and 35 °C, the effects of temperature on the CA enzyme activity detection were investigated. As described in Section 2.4.5, the CA enzyme activity values for this experiment were set in the range of 100–2000 U mL^−1^ for the enzymatic reaction curve preparation. In the water bath, the Tris–HCl buffer was previously equilibrated for at least 30 minutes at the setting temperature and the enzymatic reaction curves for different Tris–HCl buffer concentrations were recorded according to Section 2.3.4. The effects of the temperatures on the CA enzyme activity detection could be evaluated by the analysis of the reaction curves obtained under different temperatures.

### 2.5 Establishment of the enzyme activity standard curve

After establishment of the colorimetric reaction system and optimization of the colorimetric reaction system, the standard curve for the CA enzyme activity was obtained under the conditions confirmed in the previous work in Sections 2.3 and 2.4. The recorded enzymatic reaction kinetic curves were obtained with 14 enzyme activity gradients (25–2000 U mL^−1^), which included 25, 100, 200, 300, 400, 500, 600, 800, 1000, 1200, 1400, 1600, 1800, and 2000 U mL^−1^ of CA enzyme standard solutions.

**Fig. 2:**
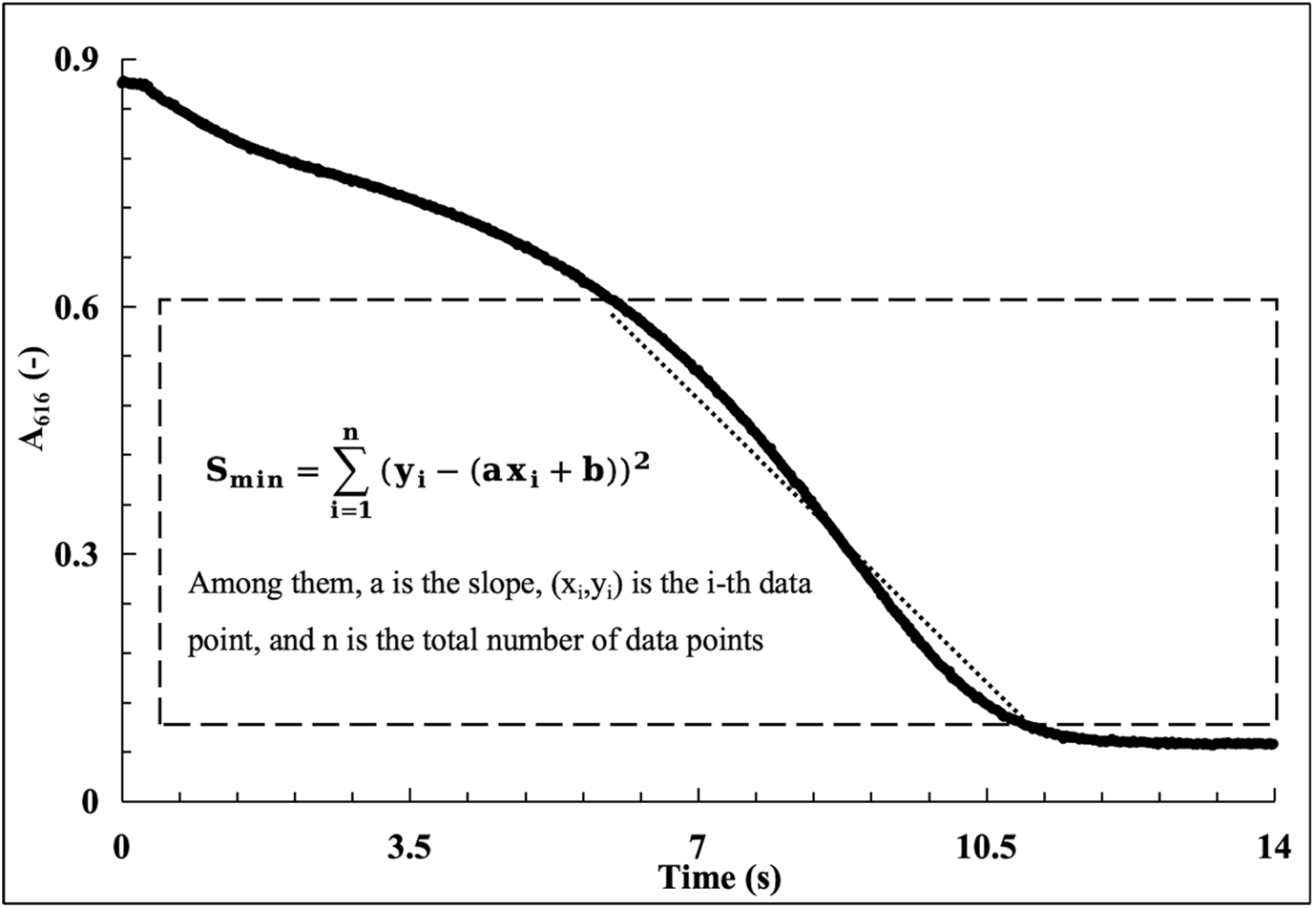
The data analysis of the enzymatic reaction dynamic curve by the least-squares method.

In order to establish the functional equation of the CA enzyme activity assay, two techniques were employed for the data analysis of the acquired enzymatic reaction dynamic curve. Just as shown in Fig. 2, in method 1 (the least square method), all data points and the associated detection times *T* (s) were recorded during the period of continuous absorbance value decline from 0.6 to 0.1. The regression analysis approach of least squares, which determines the best-fit straight line, was applied with time (*T*) as the horizontal coordinate and ABS as the vertical axis. Finding a set of parameters that minimizes the sum of squares of the errors between the observed values of *y*_*i*_ and the predicted values of the function of *f*(*x*_*i*_), as indicated by the following equations, is the aim of the least squares technique:
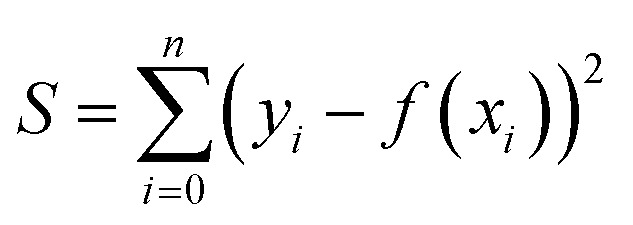


The final linear fit equation was then obtained by processing a subset of the data points:*y* = *kx* + *b*where the slope *k* represents the enzymatic reaction rate of the CA standards or test samples. Using slope *k* as the horizontal coordinate and the corresponding CA activity (U mL^−1^) as the vertical coordinate, linear regression analysis was carried out to create the standard curve.

**Fig. 3:**
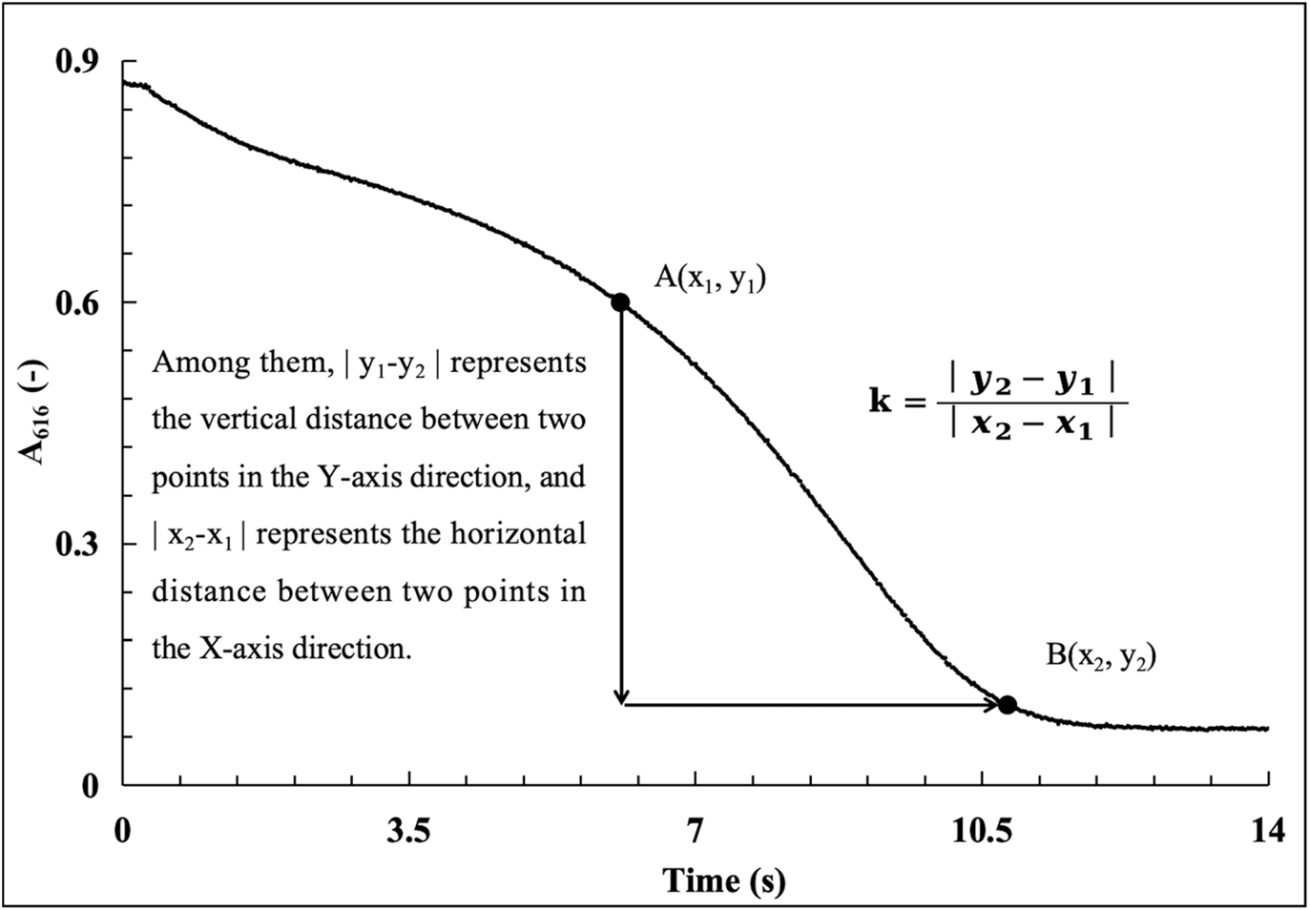
The data analysis of the enzymatic reaction dynamic curve slope calculation using the two-points method.

Method 2 (slope calculation using the two-points method) is shown in Fig. 3. Here, the slope *k* is determined using the formula and the associated time (*x*) can be determined by choosing the two absorbance eigenvalues (*y*) as 0.6 and 0.1. By recording these two sets of data as the coordinate coordinates (*x*_1_, *y*_1_) (*x*_2_, *y*_2_), and then utilizing these two known coordinate points, the final linear fit equation can then be obtained by processing a subset of the data points:*y* = *kx* + *b*where the enzymatic reaction rate of the CA standards or test samples is represented by the slope *k*. Using slope *k* as the horizontal coordinate and the corresponding CA activity (U mL^−1^) as the vertical coordinate, linear regression analysis was carried out to create the standard curve.

According to method 1 and method 2 mentioned above, the CA enzyme activity standard curve can be obtained in the form of a functional equation of slope *k* values *vs.* CA enzyme activities and with linearity of certain values, which can then be used for the applicability comparison and evaluation of these two methods.

### 2.6 Trace detection experiment

#### 2.6.1 Effect of the enzyme sample addition volume on the LOD

In this study, the LOD of the CA enzyme measurement was positively adjusted by increasing the addition volume of CA solution in the enzyme reaction system. The CA concentration gradient was set in a range of values below 30 U mL^−1^ (2 U mL^−1^, 5 U mL^−1^, 10 U mL^−1^, 15 U mL^−1^, 20 U mL^−1^, 25 U mL^−1^, and 30 U mL^−1^), with the corresponding additive CA solution volumes of 20, 40, 60, 80, 100, and 120 µL for each enzyme activity. For every volume of the solution samples, three parallel measurements were performed. The RSD was subsequently calculated to evaluate the effects of the additive CA enzyme sample solution volume on the detection limit of the measurement, which was defined as the enzyme activity value corresponding to RSD values of 10% among the parallel enzyme assay samples in this study.

The RSD is calculated as follows:
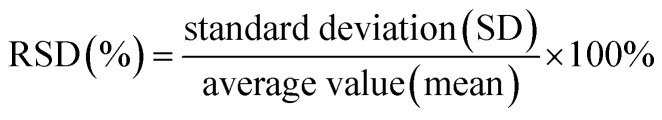


#### 2.6.2 Establishment of a standard curve for trace carbonic anhydrase

According to the results described in Section 2.6.1, the additive volume of CA sample solution was set at 120 µL, corresponding with the LOD of 2 U mL^−1^ in this study. Several CA standard solutions with a concentration gradient in the range of 2–25 U mL^−1^ were prepared and the trace CA activity standard curve was obtained according to the method described in Sections 2.3.4 and 2.5 (method 1) with 120 µL additive volume of CA standard solution for each enzyme activity concentration.

### 2.7 Methodological validation of the spectrophotometric assay for CA activity

Methodological validations in this study were carried out, including the repeatability, precision and recovery. CA enzyme sample solutions at high, medium and low concentration levels were prepared, respectively, with the concentration range of 25–2000 U mL^−1^ for the conventional test and 2–25 U mL^−1^ for the trace test. The sample of each concentration value was measured six times in accordance with Section 2.3.4, and the RSD values for each sample were calculated for repeatability validation. Precision assessment was performed through sample testing by different working time points and different operators for both conventional and trace experiments. Each sample was measured repetitively during 3 working days with 3 parallel measurements for each time. The precision validation was assessed by the RSD values from repetitive experiments. Regarding the recovery validation, CA sample solutions with low, medium and high concentration levels were set in the range of 25–2000 U mL^−1^ for the conventional test and 2–25 U mL^−1^ for the trace test. The concentration value of each sample was set to be composed from the reference concentration and the additive concentration. After the determination of three parallel results, the recovery value was calculated by dividing the value of the detection results after deducting the reference concentration and the additive concentration.

### 2.8 Medical application of the spectrophotometric method for the detection of CA activity

#### 2.8.1 Detection of the CA enzyme activity in human blood samples

The Medical Ethics Committee of Zunyi Medical University has approved this experimental protocol.

Fresh human blood samples were collected in anticoagulated tubes and vortexed and then mixed and stored at 4 °C after being diluted twofold, fourfold, sixfold and eightfold with saline containing 0.5% sodium citrate. The CA enzyme activity of each diluted sample was measured according to Section 2.3. Moreover, to evaluate the effects of Hb on the CA enzyme activity measurement, a series of CA sample solutions with varying ratios of Hb concentration to CA enzyme activity were prepared and the ratios were set to be 450 : 0, 450 : 5, 450 : 10, 450 : 30, 450 : 60, 450 : 100, and 450 : 150 (U : mg Hb), respectively. The CA activity of each sample was measured in triplicate and the average results of the CA samples with different ratios were statistically analysed with SSPR software.

#### 2.8.2 Pharmacodynamic determination of CA enzyme activity in rats

Three male Sprague-Dawley rats weighing 250–300 g were chosen and allowed to acclimate for 20 minutes following systemic heparinization (1000 U kg^−1^). After being anesthetized with pentobarbital at 30 mg per g body weight, the experimental animals were intubated in both femoral artery and vein using heparinized catheters. The right femoral vein was cannulated for the intravenous infusion of CA enzyme at a dosage of 4 mL and a rate of 1 mL min^−1^, and the right femoral artery was cannulated for blood sampling to measure the CA enzyme activity. After administration of the CA enzyme solution (background), the blood samples were collected from the experimental rats at several time points of 0, 2, 6, 8, 10, 12, 14, 16, 18, 20, 25, 30, 45, and 60 minutes. Subsequently, they were centrifuged at 2500 rpm (centrifugal force 596×*g*) for 10 minutes for the separation of the resulting plasma, which was used for the CA enzyme activity measurement. After the investigation during the experimental process, the pharmacodynamic curve of the CA enzyme (enzyme activity U mL^−1^*vs.* time minute) was obtained and the half-life of the CA enzyme in experimental rat plasma was calculated. The sample enzyme activity during the experiment can be determined by the constant standard curve or trace standard curve according to the actual situation of the samples.

## 3. Result

### 3.1 Establishment of the colorimetric reaction system

#### 3.1.1 Measurement wavelength investigation

The detection wavelength was determined based on the pH-responsive color change of BTB. A 0.015 g per L BTB solution was prepared in Tris–HCl buffer (pH 6.25, 6.75, 7.25, 7.75, 8.25; 0.02 mol L^−1^), and its UV-visible spectra were scanned over the wavelength range of 250–750 nm. Three distinct absorption peaks were observed at 307 nm, 430 nm, and 616 nm. With increasing pH, the 307 nm and 430 nm peaks exhibited a redshift, accompanied by a decrease in absorbance at 307 nm and an increase at 430 nm. In contrast, the 616 nm peak maintained a stable position, and its absorbance increased significantly with pH—this stability and pH sensitivity made 616 nm the optimal detection wavelength.

#### 3.1.2 Correlation between the concentration/pH and absorbance of the BTB solution

**Fig. 4:**
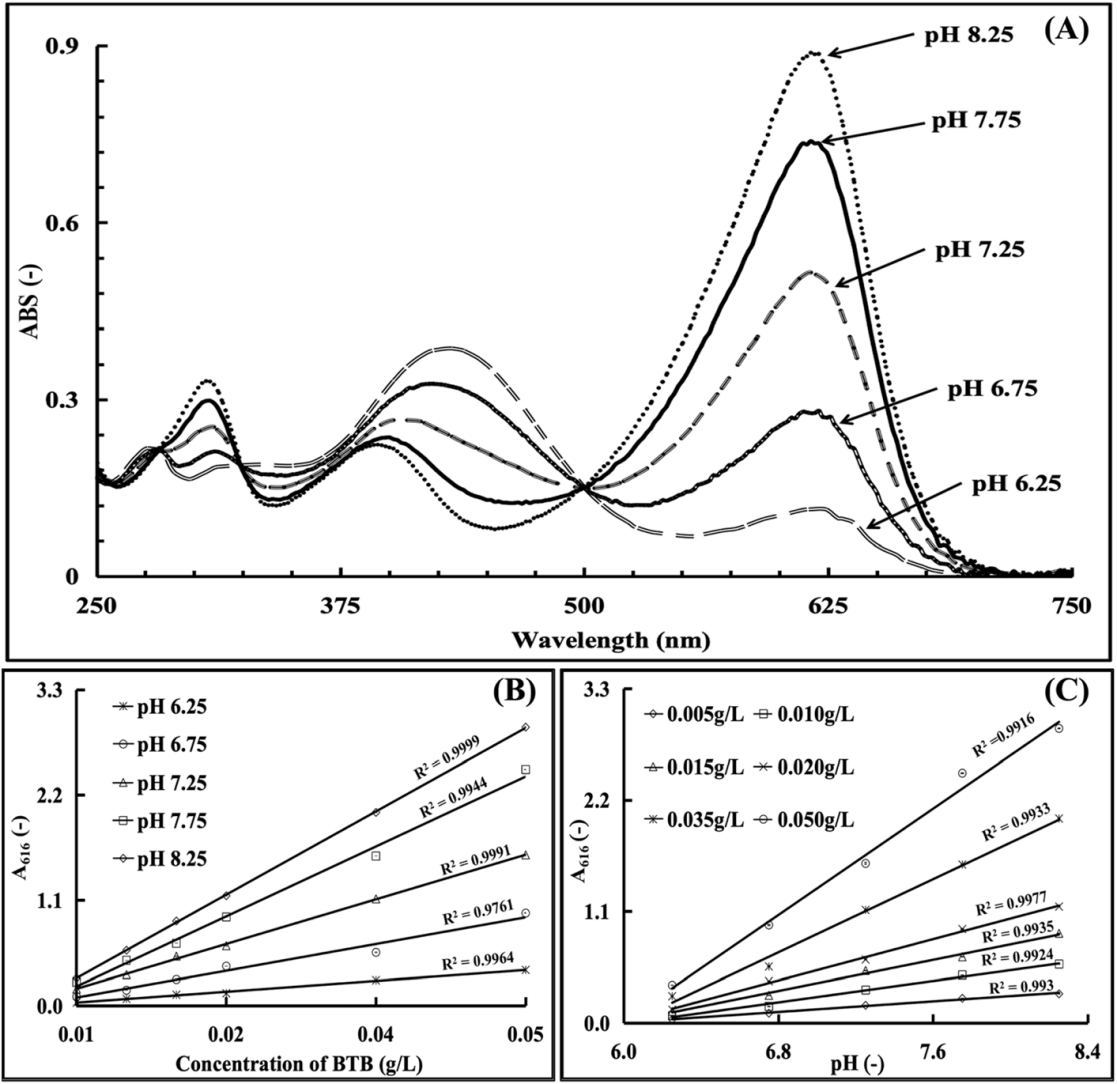
Establishment of the colorimetric reaction system ((A) UV-spectra of BTB in the Tris–HCl buffer with different pH values; (B) correlation between the concentration and absorbance of BTB solution; and (C) correlation between the pH and absorbance of BTB solution).

To confirm BTB's suitability as a photometric probe, the linearity between the BTB concentration (0.01–0.05 g L^−1^) and absorbance at 616 nm was tested across the pH range of 6.25–8.25 (Fig. 4B), yielding *R*^2^ > 0.99 for all pH values. Additionally, the linearity between the BTB solution pH and absorbance at 616 nm (0.005–0.050 g per L BTB) was verified with *R*^2^ > 0.99 (Fig. 4C), confirming BTB's reliability for pH-dependent CA activity monitoring.

### 3.2 Optimization of the reaction system for CA enzyme activity measurement

#### 3.2.1 Assessment of the BTB effect on the detection of the CA enzyme activity

**Fig. 5:**
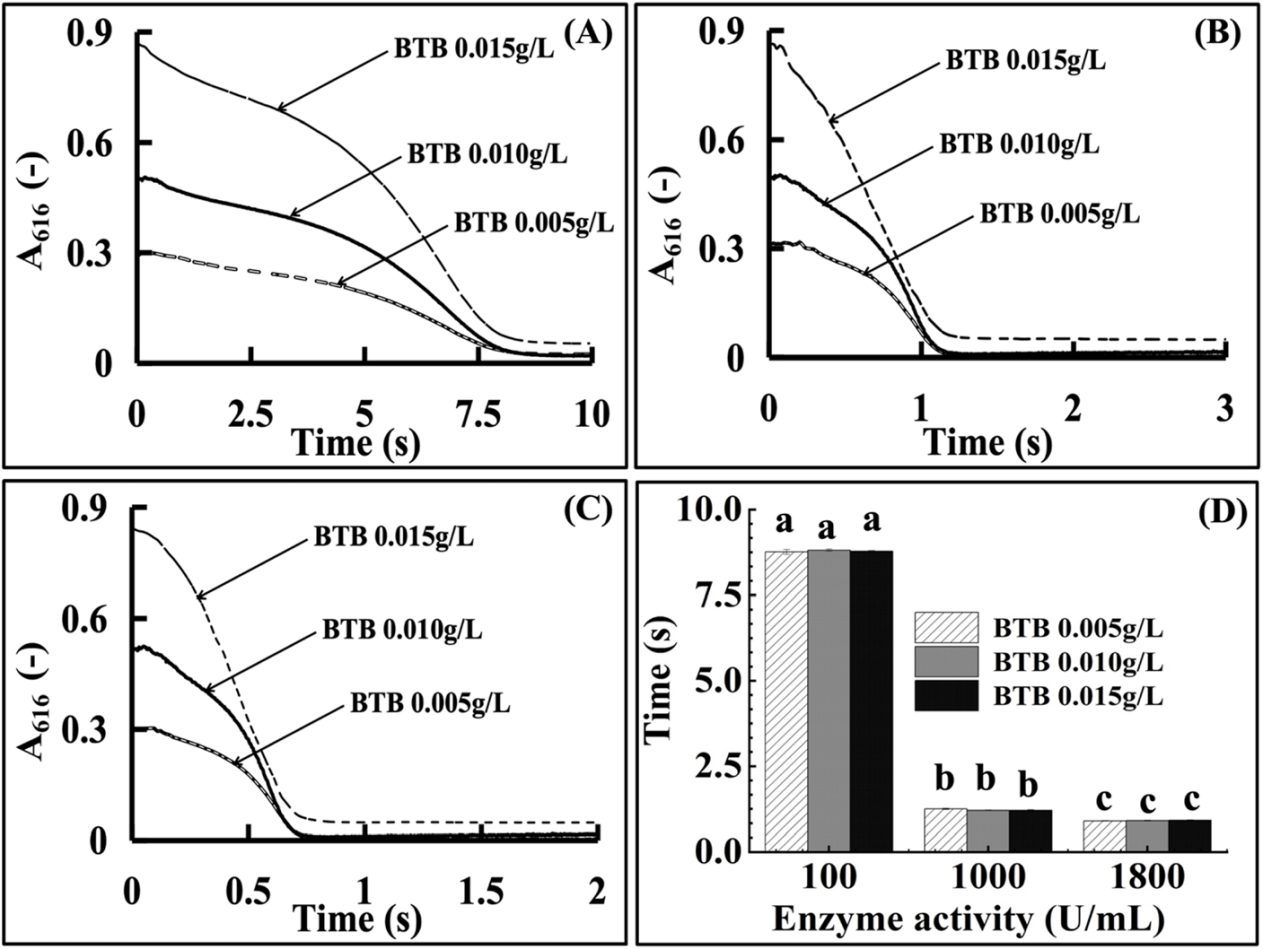
The effect of the photometric probe BTB on the detection of the CA enzyme activity ((A) 100 U mL^−1^ enzyme kinetic curve at various BTB concentrations; (B) 1000 U per mL enzyme kinetic curve at various BTB concentrations; (C) 1800 U per mL enzyme kinetic curve at various BTB concentrations; and (D) statistical evaluation of the enzymatic reaction time at various BTB concentrations). BTB of the investigated concentrations exhibited no significant (*p* > 0.05) effects.

BTB concentrations of 0.005, 0.010, and 0.015 g L^−1^ were tested. Enzymatic dynamic curves for 100, 1000, and 1800 U per mL CA (Fig. 5A–C) and reaction duration comparisons (Fig. 5D) showed no statistical differences in the reaction duration between groups (*p* > 0.05). This indicated that BTB (0.005–0.015 g L^−1^) had no inhibitory effect on the CA activity, with 0.015 g L^−1^ selected for sufficient absorbance signal.

#### 3.2.2 Effect of the Tris–HCl buffer concentration on the CA enzyme activity detection

**Fig. 6:**
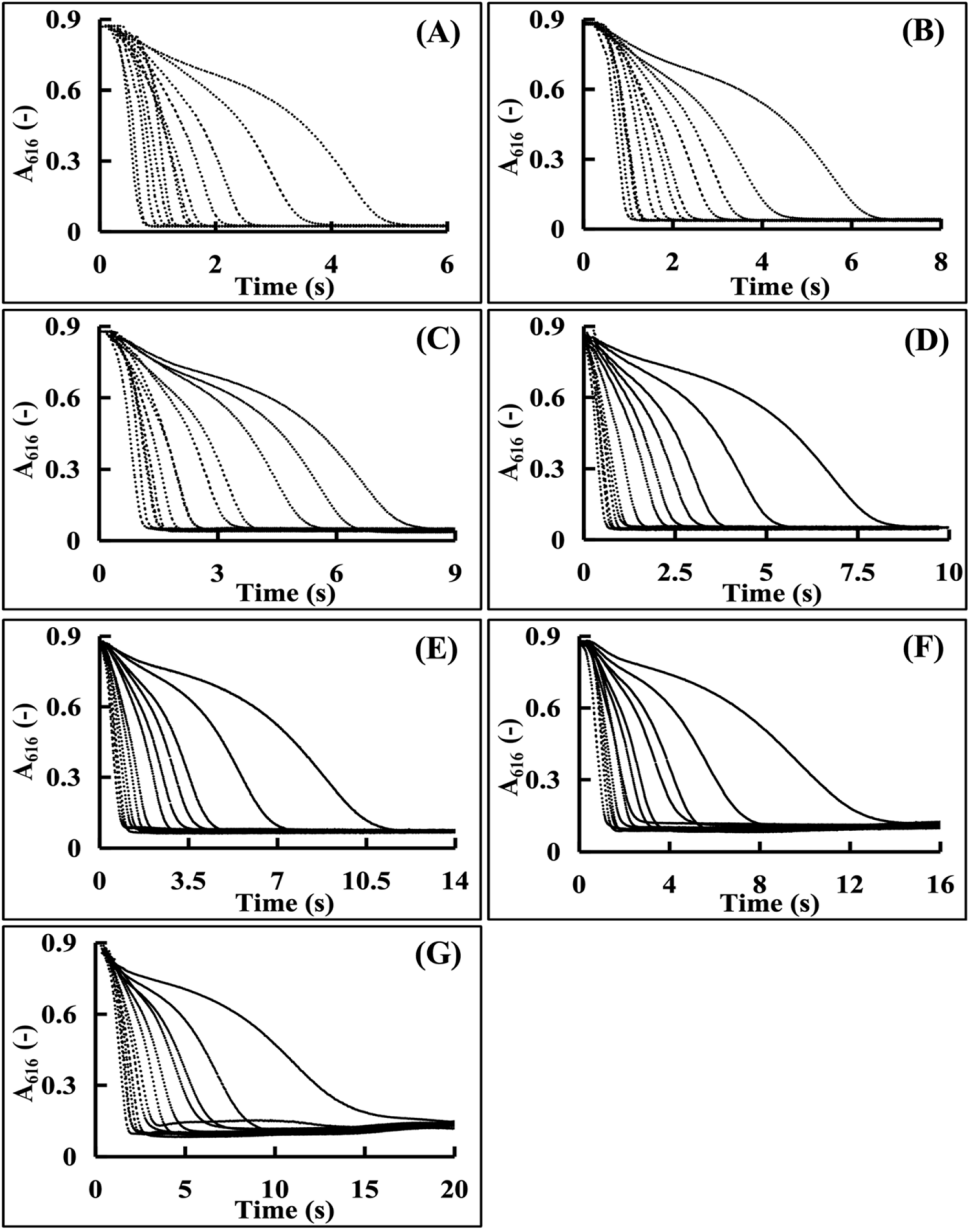
Kinetic curves by different concentrations of the Tris–HCl buffer solutions (the curves from right to left correspond to enzyme activities of 100, 200, 300, 400, 500, 600, 800, 1000, 1200, 1400, 1600, 1800, and 2000 U mL^−1^, respectively). (A) 0.015 mol L^−1^. (B) 0.020 mol L^−1^. (C) 0.025 mol L^−1^. (D) 0.028 mol L^−1^. (E) 0.030 mol L^−1^. (F) 0.035 mol L^−1^. (G) 0.040 mol L^−1^.

Tris–HCl buffer concentrations (0.015, 0.02, 0.025, 0.03, 0.035, 0.04 mol L^−1^) were evaluated using 100–2000 U per mL CA. Fig. 6 shows the complete kinetic curves at the concentration range of 0.015–0.03 mol L^−1^. However, higher concentrations (Fig. 6F and G) caused significant baseline fluctuations, destabilizing the reaction system. Within the tested range, the reaction duration increased with Tris–HCl concentration due to the inhibited hydrogen ion release.

Lower concentrations reduced the buffering capacity, accelerating the reactions and decreasing the curve discrimination, hindering accurate H^+^ monitoring. Thus, 0.028 mol L^−1^ (mid-range value) was chosen for reliable CA measurement.

#### 3.2.3 Effect of temperatures on the CA enzyme activity detection

**Fig. 7:**
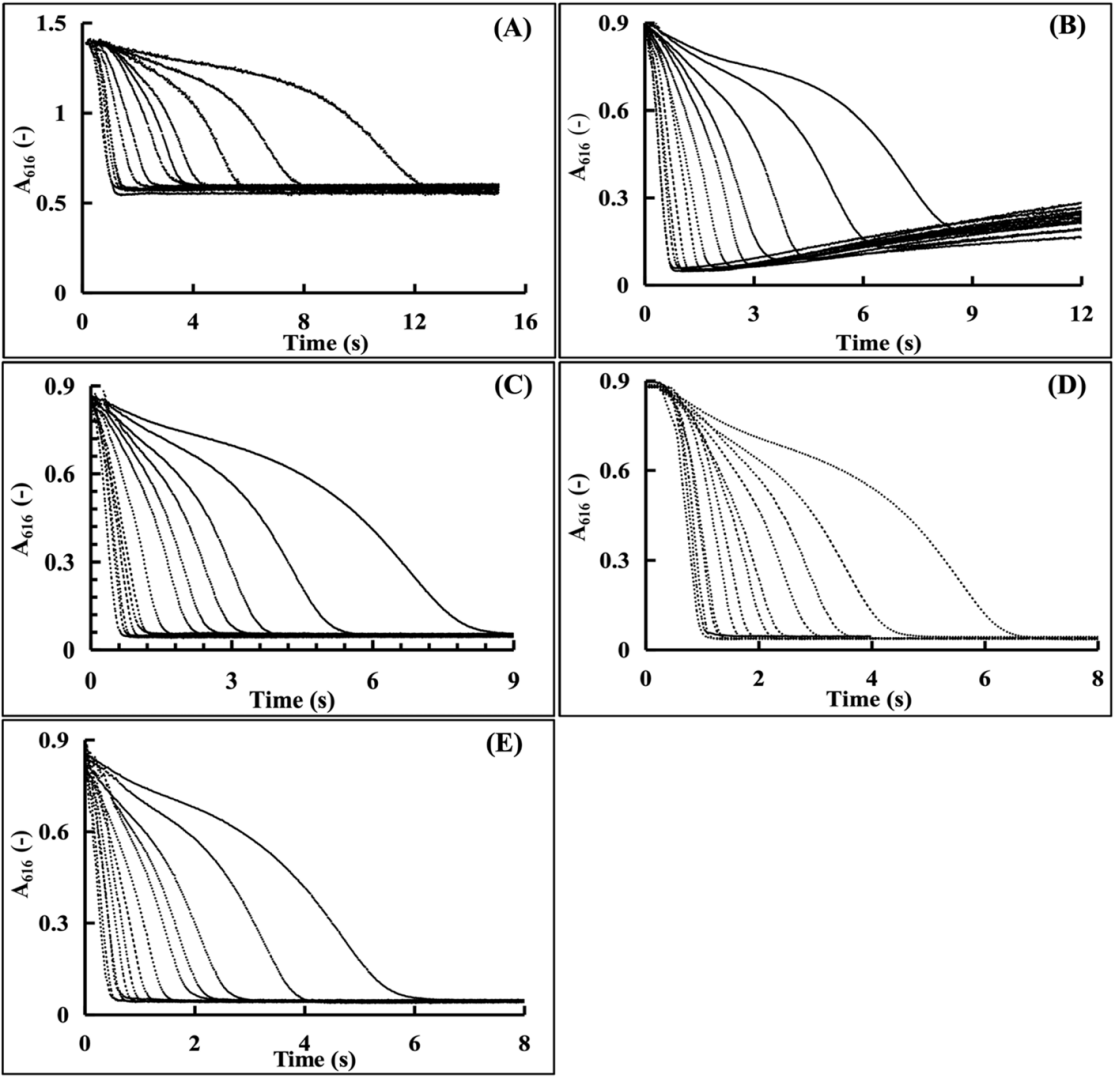
Kinetic curves of the CA enzyme activity determined by the Tris–HCl buffer at different temperatures (the curves from right to left correspond to the enzyme activities of 100, 200, 300, 400, 500, 600, 800, 1000, 1200, 1400, 1600, 1800, and 2000 U mL^−1^, respectively. respectively). (A) 0 °C; (B) 15 °C; (C) 20 °C; (D) 25 °C; (E) 35 °C.

Enzymatic curves were recorded at 0, 10, 15, 20, 25, 30, and 35 °C (Fig. 7). Unstable baselines were observed at <15 °C, attributed to incomplete CO_2_ hydration (partial CA inactivation) and pH elevation (increased BTB p*K*_a_ at low temperatures).^48^ Stable, complete curves were obtained at 20–35 °C, with shorter reaction durations at higher temperatures (consistent with enzyme kinetics). To avoid the reduction of data discrimination from excessive reaction speeds, 20 °C was selected as the assay temperature.

In summary, as the photometric probe in this study, BTB presents excellent spectrophotometric characteristics at the wavelength of 616 nm and exerted no significant inhibitory effects on the CA activity detection in the chromogenic system, thereby ensuring the precision and reliability of the studied detection method. The conditional indexes for the subsequent methodology validation were confirmed as follows: 0.015 g per L BTB concentration, 616 nm wavelength value, 0.028 mol per L of Tris–HCl buffer concentration, and 20 °C temperature, respectively.

### 3.3 Establishment of a conventional enzyme activity standard curve

**Fig. 8:**
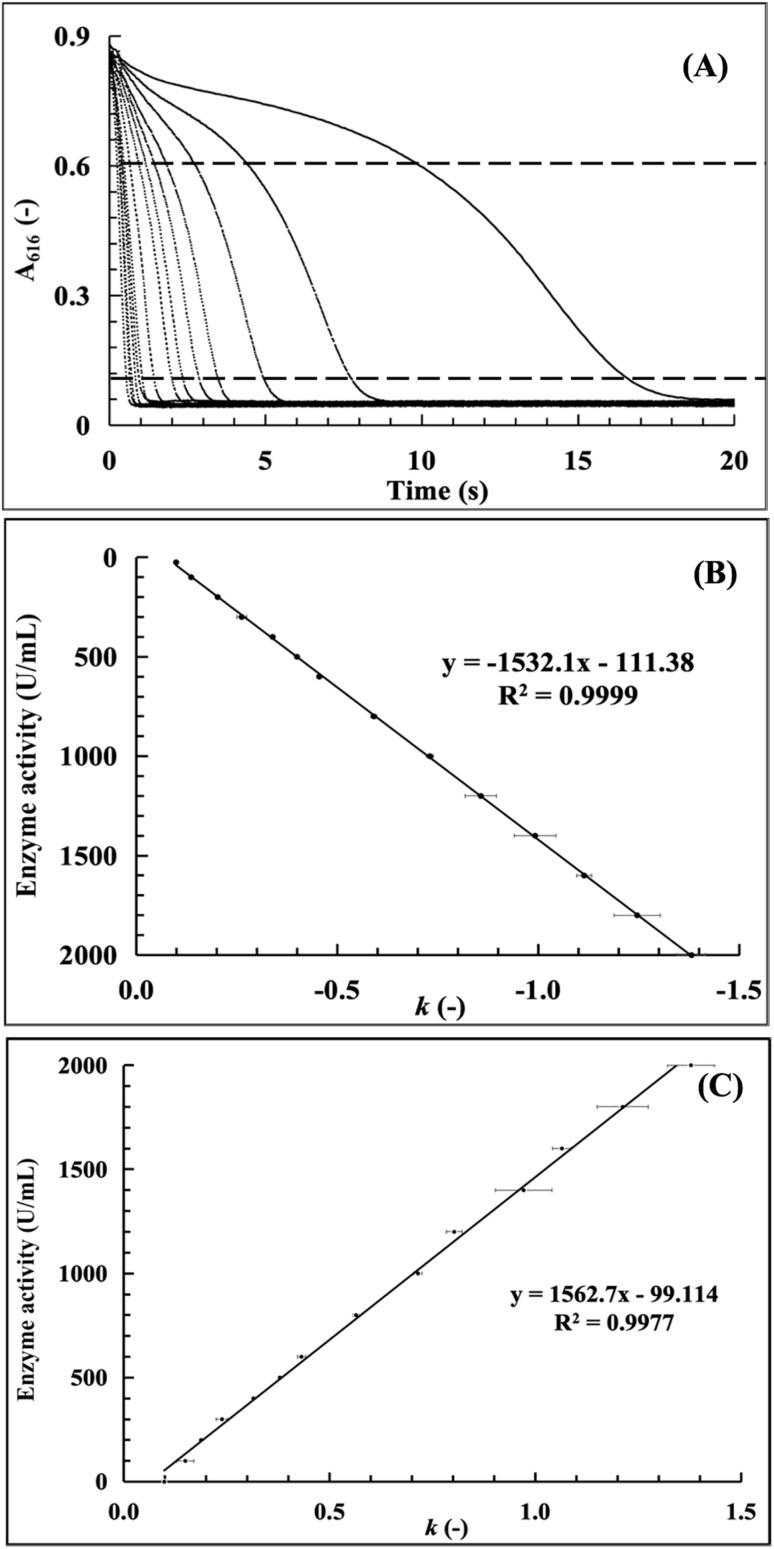
Establishment of the conventional enzyme activity standard curve (25–2000 U mL^−1^) ((A) enzymatic reaction kinetics curves in a CA concentration range of 25–2000 U mL^−1^; (B) the standard curve established by the least-squares method; and (C) the standard curve established by the two-points method).

Enzymatic reaction dynamic curves were acquired under the optimized conditions (BTB: 0.015 g L^−1^; wavelength: 616 nm; Tris–HCl: 0.028 mol L^−1^; temperature: 20 °C) using CA standard solutions (25–2000 U mL^−1^). Representative curves (Fig. 8A) clearly distinguished between different CA activities, illustrated the full reaction process (and its correlation with CA activity for quantification), and revealed two key trends: the reaction time span increased with decreasing CA activity (higher activity shortened duration), and the absorbance at 616 nm decreased rapidly in the early stage (high CO_2_ hydration rate) before slowing gradually until completion (due to substrate depletion).

Standard curves for CA activity quantification were generated *via* two-slope calculation methods (Fig. 8B and C): the least squares method, which minimized squared errors to fit a high-accuracy linear model reflecting overall data trends, and the two-point method, which simplistically calculated slopes between *A*_616_ values of 0.6 and 0.1 but ignored full dataset trends (for more details, see Tables S1 and S3, SI). Both methods yielded *R*^2^ > 0.99 across 25–2000 U per mL CA, confirming strong linearity between the CA activity and slope (*k* values); the least squares method was prioritized for its superior accuracy, while the two-point method was noted for its simplicity but reduced representativeness.

### 3.4 Detection for trace amount of CA enzyme activity

It was notable that the value range of the standard curve for CA activity measurement was set with the lowest value of 25 U mL^−1^, which could be considered as the LOD of the optimized enzymatic reaction system. However, according to the characteristics of the enzymatic reaction system, the addition of a low enzyme amount resulted in an extended duration of the enzymatic reaction. When approaching the lower concentration limit of the detection range, the addition of trace CA enzyme concentration will reduce the stability and reliability of the enzymatic reaction process, thus leading to the repeatability and reliability descending of the reaction dynamic curve.

#### 3.4.1 Effect of enzyme sample addition volume on LOD

**Fig. 9:**
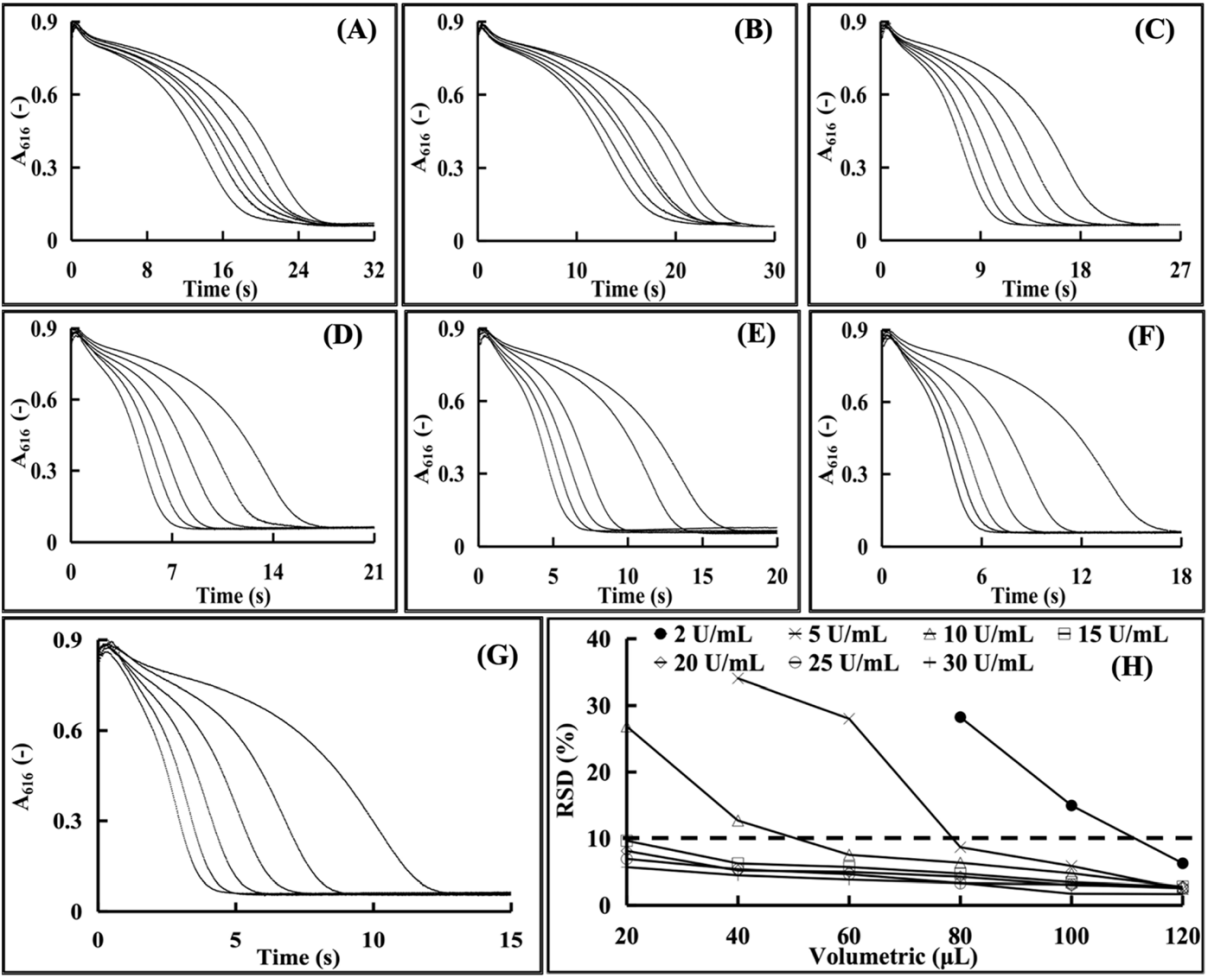
The influence of the addition volume of enzyme samples on the LOD (A): 2 U mL^−1^. (B): 5 U mL^−1^. (C): 10 U mL^−1^. (D): 15 U mL^−1^. (E): 20 U mL^−1^. (F): 25 U mL^−1^. (G): 25 U mL^−1^ (the curves, starting from the rightmost one, respectively correspond to enzyme sample volumes of 20 µL, 40 µL, 50 µL, 80 µL, 100 µL, and 120 µL). (H): the relative standard deviation (RSD) under different enzyme sample volumes (the curves, starting from the rightmost one, respectively correspond to enzyme addition volumes of 20 µL, 40 µL, 50 µL, 80 µL, 100 µL, and 120 µL).

The impact of varying addition volumes on the *A*_616_ absorbance change in the CA enzyme activity assay procedure was investigated while keeping the concentration constant of added enzyme activity, the results of which are shown in Fig. 9(A–G). At each investigated CA concentration, the time span values of the dynamic curves decreased with increasing CA addition volume, demonstrating the much faster reaction rates caused by the higher CA amounts in the enzymatic assay reaction system. The correlations between the CA addition volume and RSD values of the results at each investigated CA concentration are presented in Fig. 9(H). Results showed that within the range of the experimental CA addition volume, to reach the LOD standard of less than 10% RSD value, the addition volume increased with decreasing CA enzyme concentration. For example, at the CA concentration of 2 U mL^−1^, the addition volume should be nearly 120 µL to make the RSD value less than 10%, suggesting the more CA amount addition for the stabilization of the enzymatic assay system. Therefore, the LOD of the CA activity measurement could be adjusted through varying the addition volume of CA sample solution, which would improve the accuracy and applicability for CA enzyme determining, thereby facilitating further research and practical implementation in related fields.

#### 3.4.2 Establishment of a standard curve for trace CA enzyme amount detection

**Fig. 10:**
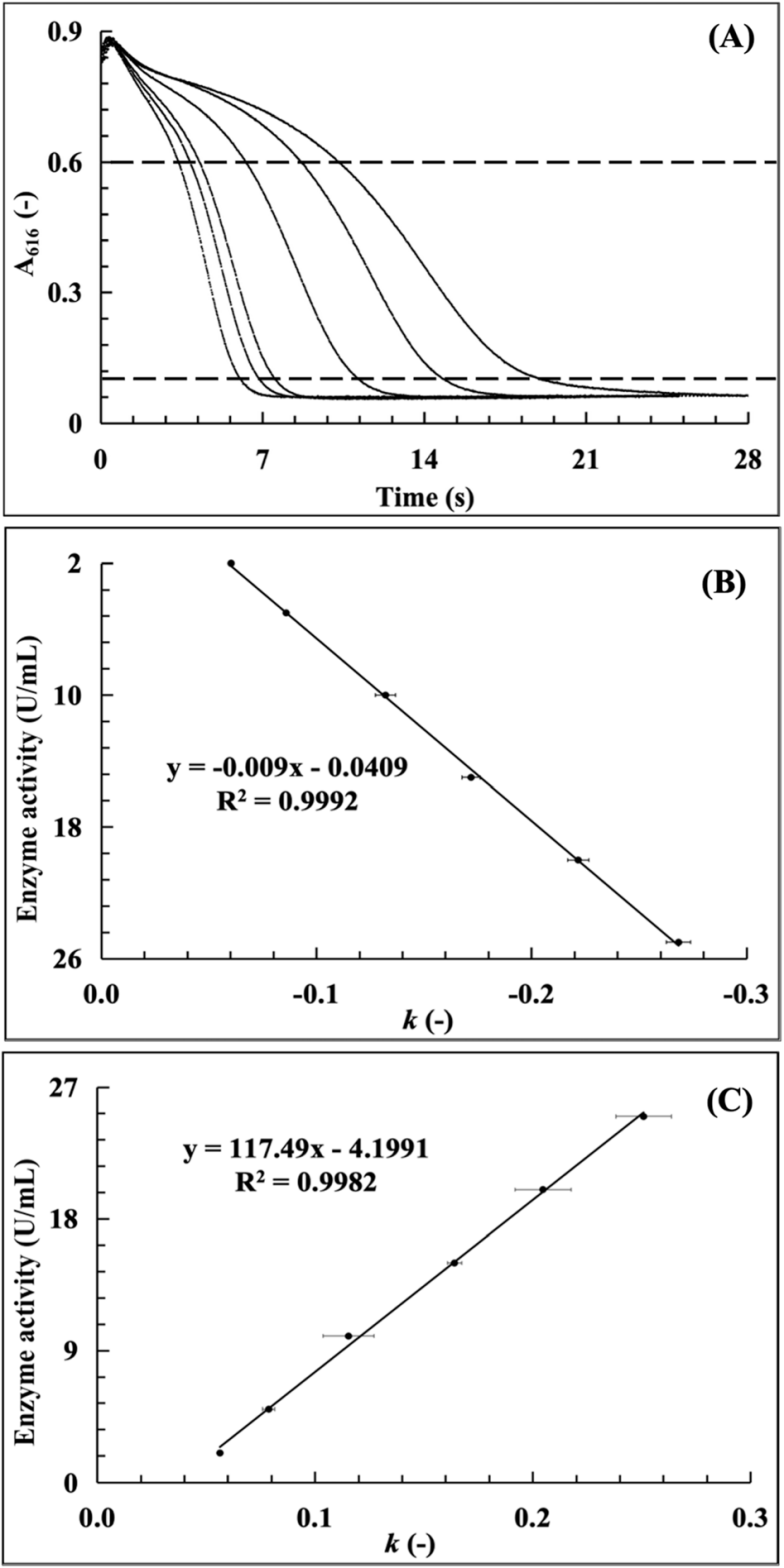
Establishment of a standard curve for the trace amounts of CA (2–25 U mL^−1^) ((A) enzymatic reaction kinetics curves in the CA concentration of 2–25 U mL^−1^; (B) the standard curve established by the least-squares method; and (C) the standard curve established by the two-points method).

According to the results from the effects of the CA addition volume on the LOD values, the CA enzyme addition volume for the trace experimental standard curves were determined at 120 µL, which could not only ensure the stability of the enzymatic assay reaction system, but also reduce the LOD to below 2 U mL^−1^. The trace CA enzymatic reaction dynamic curves in the CA concentration range of 2–25 U mL^−1^ are presented in Fig. 10(A), from which the dynamic curves were arranged from left to right and clearly distinguished according to the order of enzyme activity values. The trace experimental standard curves calculated from Fig. 10(A) are shown in Fig. 7(B) with the linearity value of 0.9992, suggesting the highly significant linear correlation between the trace amount of CA enzyme and the slope. The standard curves for determining the trace CA activity by the least squares method and the two-point method are shown in Fig. 10(B) and (C), respectively (for more details, see Tables S3 and S4, SI). It can be seen that the linear values of both standard curves within the experimental range are higher than 0.99, among which the linear relationship of the least squares method is even higher.

The adjustment of the LOD for the CA activity and establishment of the trace experimental standard curve exhibited an effective technological approach for the CA micro-detection. It could extend the application of the studied method in the accurate determination of CA activity during physiological and pathological processes, especially with respect to the screening, diagnosis and quantification of the CA-related cancer markers.

### 3.5 Methodological validation

#### 3.5.1 Repeatability

The repeatability validation of the studied method was carried out *via* measurements for both conventional and trace CA concentrations at the low, medium and high levels with six parallel tests for each sample. The results are shown in Tables 1 and 2, from which the RSD values of the experimental samples were all less than 3%, suggesting the small dispersion degree and reliable repeatability of the parallel tested values.

**Table 1:** Repeatability results for the conventional CA activity determination

Sample	Parallel number	Tested sample (U mL^−1^)	Average results (U mL^−1^)	RSD (%)
Sample 1	1	173.71	176.41	2.46
	2	171.43		
	3	173.86		
	4	180.14		
	5	176.43		
	6	182.86		
Sample 2	1	1070.29	1078.33	2.19
	2	1096.57		
	3	1100.29		
	4	1085.71		
	5	1081.71		
	6	1035.43		
Sample 3	1	1875.57	1861.98	0.89
	2	1882.57		
	3	1863.57		
	4	1836.14		
	5	1853.00		
	6	1861.00		

**Table 2:** Repeatability results for the trace CA activity determination

Sample	Parallel number	Tested sample (U mL^−1^)	Average results (U mL^−1^)	RSD (%)
Sample 1	1	4.87	4.95	2.73
	2	5.09		
	3	5.14		
	4	4.90		
	5	4.79		
	6	4.92		
Sample 2	1	13.71	14.15	2.21
	2	14.20		
	3	13.83		
	4	14.29		
	5	14.31		
	6	14.53		
Sample 3	1	19.50	19.44	1.17
	2	19.74		
	3	19.27		
	4	19.09		
	5	19.53		
	6	19.50		

#### 3.5.2 Precision

The precision validation was performed through measurements for both conventional and trace CA concentrations at the low, medium and high levels with three intraday detections for each sample and the results are shown in Tables 3 and 4. It can be seen that the RSD values from the tested results by different operators and at different operation days were all below 3% for the investigated samples. The results of this experiment indicate that this studied method for CA activity detection was rarely influenced by differences in time and personnel.

**Table 3:** Precision experiments for the CA conventional activity determination

Day	Intraday mean value (U mL^−1^)	Interday mean value (U mL^−1^)	RSD (%)
Day 1	173.10	175.30	2.23
Day 2	173.00		
Day 3	179.81		
Day 1	1095.52	1084.06	1.35
Day 2	1089.05		
Day 3	1067.62		
Day 1	1868.00	1863.98	0.67
Day 2	1873.90		
Day 3	1850.05		

**Table 4:** Precision experiments for the CA trace activity determination

Day	Intraday mean value (U mL^−1^)	Interday mean value (U mL^−1^)	RSD (%)
Day 1	4.84	4.92	2.80
Day 2	5.08		
Day 3	4.83		
Day 1	14.42	14.36	2.90
Day 2	13.91		
Day 3	14.74		
Day 1	18.70	18.88	0.97
Day 2	19.07		
Day 3	18.86		

#### 3.5.3 Recovery

The results of the spiked recoveries of the samples at three distinct concentration levels for both conventional and trace CA amounts are presented in Tables 5 and 6, with three parallel measurements for each sample. The average recovery rates of these investigated experimental samples were all among the range of 100% ± 2%, which indicated that this studied method could accurately determine the CA enzyme activity with small errors.

**Table 5:** Recovery experiments of the conventional CA activity

Basic enzyme activity (U mL^−1^)	Spiked enzyme activity (U mL^−1^)	Tested enzyme activity (U mL^−1^)	Tested spiked enzyme activity (U mL^−1^)	Recovery rate (%)	Average recovery rate (%)
176.41	100	277.43	101.02	101.02	98.26 ± 1.69
		272.86	96.45	96.45	
		273.71	97.31	97.31	
	1000	1184.57	1008.17	100.82	99.74 ± 2.58
		1192.29	1015.89	101.59	
		1144.43	968.03	96.80	
	1800	1910.86	1734.46	96.36	98.08 ± 1.84
		1939.14	1762.74	97.93	
		1975.71	1799.31	99.96	

**Table 6:** Recovery experiments of the trace CA activity

Sample enzyme activity (U mL^−1^)	Spiked enzyme activity (U mL^−1^)	Tested enzyme activity (U mL^−1^)	Tested spiked enzyme activity (U mL^−1^)	Recovery rate (%)	Average recovery rate (%)
4.95	5	10.01	5.06	101.20	102.2 ± 1.69
		10.19	5.24	104.80	
		9.98	5.03	100.6	
	10	15.22	10.27	102.70	101 ± 1.48
		14.94	9.99	99.90	
		14.99	10.04	100.40	
	15	20.18	15.23	101.53	101.69 ± 0.70
		20.11	15.16	101.07	
		20.32	15.37	102.47	

### 3.6 Application of the method of CA activity detection by UV spectrophotometry

#### 3.6.1 Detection of the CA enzyme activity in human blood samples

Human blood samples with a series of dilutions (from 1 to 8 times) were tested with the conventional standard curve and the results are shown in Table 7. The results with lower data dispersion were obtained from three parallel measurements. The RSD values of the results were all below 5% under each dilution time of the human blood sample, indicating the stability and accuracy of the tested results. Moreover, the RSD values of the results from the tested samples with different dilution times were less than 3%, suggesting the applicability of this spectrophotometric method for CA activity in clinical medical testing.

**Table 7:** Detection of the CA enzyme activity in human blood samples

Dilution ratio	Enzyme activity (U mL^−1^)	Raw enzyme activity before dilution (U mL^−1^)	Mean value (U mL^−1^)	RSD (%)	Average results (U mL^−1^)	RSD (%)
1	1074.29	1074.29	1062.90	3.21	1093.50	2.18
	1089.86	1089.86				
	1024.57	1024.57				
2	554.71	1109.43	555.76	0.89		
	551.43	1102.86				
	561.14	1122.29				
4	273.14	1092.57	270.95	0.70		
	269.71	1078.86				
	270.00	1080.00				
6	185.43	1112.57	184.52	1.35		
	186.43	1118.57				
	181.71	1090.29				
8	137.43	1099.43	137.76	0.26		
	138.14	1105.14				
	137.71	1101.71				

Human blood is rich in hemoglobin (Hb), a red blood cell component that collaborates with carbonic anhydrase (CA) in respiratory metabolism and acid–base balance. Thus, Hb interference is one of the key issues in the measurement of CA activity in clinical blood tests.

This study prepared samples with fixed CA activity (450 U mL^−1^) and varying Hb concentrations (0–150 g L^−1^, ratios of 450 : 0 to 450 : 150; Fig. 11) to evaluate the Hb impact. Statistical analysis (*p* > 0.05) showed that all measured CA activities remained at 450 U mL^−1^, even with the Hb concentrations exceeding normal physiological levels (no dilution). This confirms the assay's resistance to Hb interference, supporting reliable CA measurement in complex clinical blood samples.

**Fig. 11:**
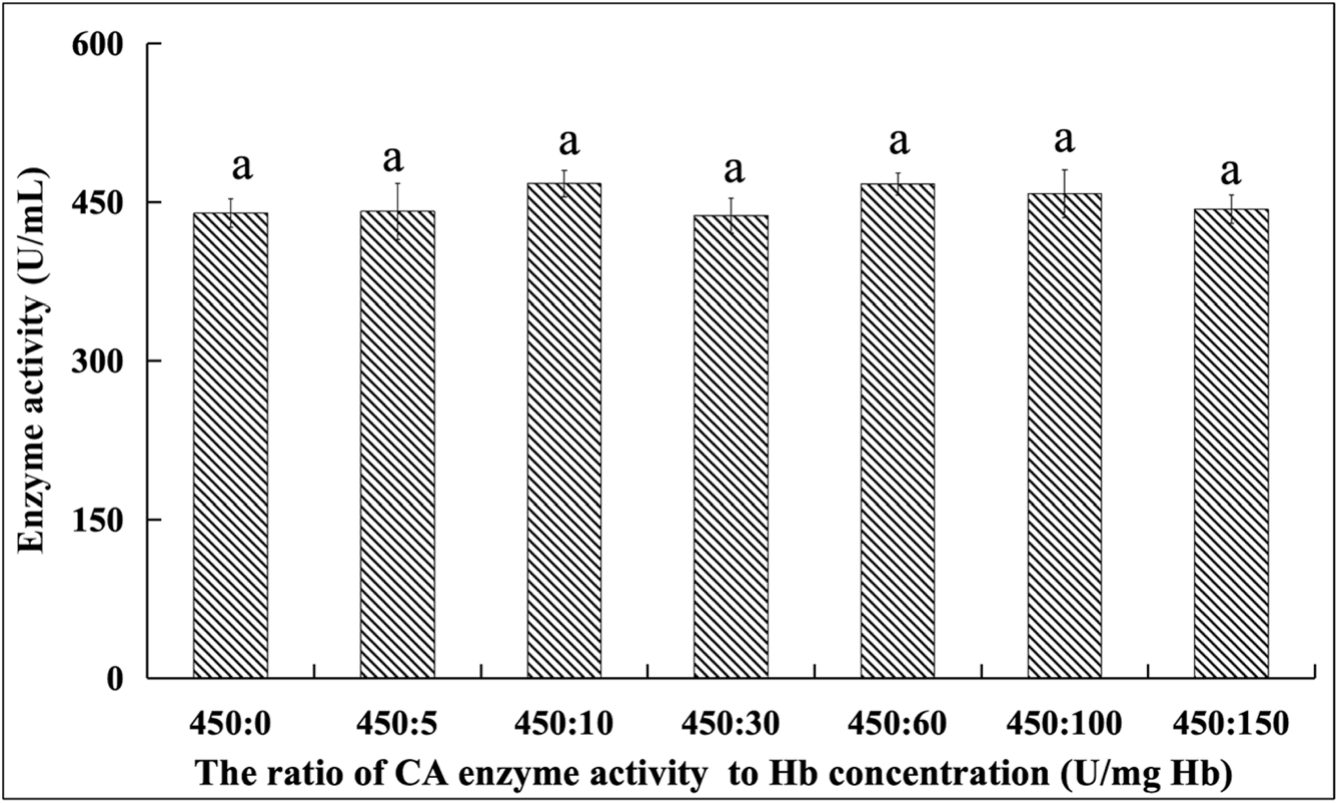
The effect of the hemoglobin concentration on the detection of the CA activity in the human blood samples (*note: the letter marks are used for statistical difference analysis. The same superscript letters (*e.g.*, “a”) indicate that there is no significant difference between groups, while different superscript letters indicate that there are significant differences between groups, reflecting the statistical difference situation of the enzyme activity measurement results of each Hb concentration group).

In conclusion, the CA activity measurement is independent of the Hb concentration under the tested ratios, verifying the UV-spectrophotometric method's accuracy for CA quantification in complex systems (*e.g.*, blood). The 616 nm detection wavelength differs from Hb's 415 nm absorption peak; however, MetHb (absorption peak at 630 nm, which is near 616 nm) may interfere in pathological states (*e.g.*, methemoglobinemia), warranting future research on methemoglobin interference mitigation. Additionally, refining this UV technique for environmental CA detection and establishing standardization will enhance method universality and support environmental CA activity analysis.

#### 3.6.2 Pharmacodynamic determination of CA enzyme activity in rats

**Fig. 12:**
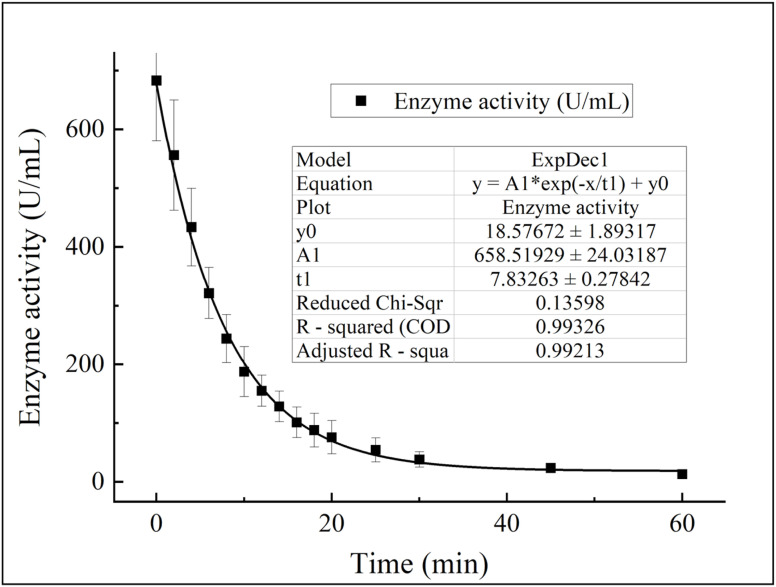
Determination of the CA activity half-life in the rats.

To mimic the *in vivo* metabolism of CA-related drugs, the pharmacokinetics of CA in rats was monitored post-intravenous administration, with the plasma CA concentrations detected *via* the studied spectrophotometric method. As shown in Fig. 12, the rat CA pharmacokinetic curve was fitted to an exponential decay model, yielding a half-life of 7.83 ± 0.27 min and a peak value of 672.47 ± 13.11 U mL^−1^; a fitting linearity of 0.99326 and adjusted *R*-square of 0.99213 validated the accuracy of these values.

Over the study duration, the plasma CA activity decreased from 700 U mL^−1^ to 10 U mL^−1^. The conventional CA standard curve tracked the concentrations during the initial 30 min (a phase of rapid fluctuation), while the trace CA standard curve was used for detection at 30–60 min post-administration (when the CA activity was in the low-concentration range) (for more details, see Tables S5 and S6, SI). Combining these two curves enabled the acquisition of an intact pharmacokinetic profile—even for trace CA concentrations—supporting more accurate pharmacokinetic analysis. Additionally, the intact curve indicated no significant negative interference from plasma components on CA detection, facilitating future clinical application of this method.

## 4 Conclusions

In this study, a highly sensitive and precise UV spectrophotometric technique based on the photometric probe BTB was successfully developed for the accurate measurement of CA activity with a LOD value as low as 2 U mL^−1^. After the establishment of the colorimetric reaction system and the optimization of the CA assay reaction system, the conventional CA standard curve was obtained with strong linearity. The LOD of the trace CA standard curve was also found to be below 2 U mL^−1^ and could even be adjusted by the additional volume of the CA sample. The results from the methodology validation demonstrated the excellent reproducibility, high precision and recoveries of the spiked samples in the 98–103% range. Finally, the applications of this studied spectrophotometric method were explored through CA measurement in human blood samples and CA pharmacokinetic research in rats. This approach demonstrates improved resistance to interferences from Hb and other plasma proteins and shows promise as a dependable analytical tool for the monitoring of CA activity in future applications such as clinical practice, pharmaceutic research and other industrial fields.

## Author contributions

Xiaoxiao Liu: writing – original draft, data analysis, conceptualization, methodology. Renci Tian: data analysis. Haoran Mi: data analysis. Wuxia Guo: writing – review & editing, supervision. Gang Chen: writing – review & editing, supervision, project administration, data curation, funding acquisition, writing.

## Conflicts of interest

The authors declare that they have no known competing financial interests or personal relationships that could have appeared to influence the work reported in this paper.

## Supplementary Material

RA-015-D5RA06480E-s001

## Data Availability

The authors declare that the data supporting the findings of this study are available within the paper and its supplementary information (SI) files. Should any raw data files be needed in another format, they are available from the corresponding author upon reasonable request. Source data are provided with this paper. Supplementary information is available. See DOI: https://doi.org/10.1039/d5ra06480e.
