# Supplementary material for: Establishment, validation and application of a spectrophotometric method for the accurate determination of carbonic anhydrase activity
Source: RSC Adv. 2025 Dec 1;15(55):47300–14. doi: 10.1039/d5ra06480e (PMC12668187; doi:10.1039/d5ra06480e)
Supplement: RA-015-D5RA06480E-s001 [file RA-015-D5RA06480E-s001.pdf]

Supplementary Information (SI) for RSC Advances.
This journal is © The Royal Society of Chemistry 2025

Electronic Supplementary Information (ESI)

for

Establishment, Validation and Application of a Spectrophotometric Method for the Accurate Determination of Carbonic Anhydrase Activity

Xiaoxiao Liu, Wuxia Guo, Gang Chen*

Table S1. Establishment of conventional enzyme activity standard curve by the least square method

enzyme activity (U/mL)	slope k_1	slope k_2	slope k_3	average slope k
25	-0.0995	-0.0994	-0.0975	-0.0988 ± 0.0011
100	-0.1349	-0.1331	-0.1408	-0.1363 ± 0.0040
200	-0.2021	-0.2046	-0.2002	-0.2023 ± 0.0022
300	-0.2747	-0.2618	-0.2494	-0.2620 ± 0.0127
400	-0.3357	-0.3365	-0.3447	-0.3390 ± 0.0050
500	-0.3977	-0.3959	-0.4045	-0.3994 ± 0.0045
600	-0.4617	-0.4529	-0.4501	-0.4549 ± 0.0061
800	-0.5914	-0.5832	-0.5980	-0.5909 ± 0.0074
1000	-0.7222	-0.7390	-0.7290	-0.7301 ± 0.0085
1200	-0.8560	-0.8958	-0.8184	-0.8567 ± 0.0387
1400	-0.9366	-0.9986	-1.0399	-0.9917 ± 0.0520
1600	-1.1027	-1.1032	-1.1346	-1.1135 ± 0.0183
1800	-1.3077	-1.2338	-1.1948	-1.2454 ± 0.0573
2000	-1.3549	-1.4200	-1.3668	-1.3806 ± 0.0347

Table S2. Establishment of conventional enzyme activity standard curve using two points method

enzyme activity (U/mL)	slope k_1	slope k_2	slope k_3	average slope k
25	0.0916	0.1016	0.1008	0.0980 ± 0.0056
100	0.1736	0.1377	0.1362	0.1491 ± 0.0212
200	0.1887	0.1908	0.1825	0.1873 ± 0.0043
300	0.2513	0.2392	0.2242	0.2382 ± 0.0135
400	0.3106	0.3125	0.3205	0.3145 ± 0.0053
500	0.3788	0.3731	0.3846	0.3788 ± 0.0057
600	0.4386	0.4202	0.4348	0.4312 ± 0.0097
800	0.5682	0.5556	0.5682	0.5640 ± 0.0072
1000	0.7042	0.7143	0.7246	0.7144 ± 0.0102
1200	0.8065	0.8197	0.7813	0.8025 ± 0.0195
1400	0.8929	1.0000	1.0204	0.9711 ± 0.0685
1600	1.0417	1.0638	1.0870	1.0642 ± 0.0226
1800	1.2821	1.1905	1.1628	1.2118 ± 0.0624
2000	1.3889	1.4286	1.3158	1.3778 ± 0.0572

Table S3. Establishment of a standard curve for trace amounts of CA by the least square method

enzyme activity (U/mL)	slope k_1	slope k_2	slope k_3	average slope k
2	-0.0602	-0.0601	-0.0604	-0.0602 ± 0.0002
5	-0.0854	-0.0862	-0.0859	-0.0858 ± 0.0004
10	-0.1288	-0.1328	-0.1353	-0.1321 ± 0.0046
15	-0.1705	-0.1715	-0.1735	-0.1718 ± 0.0015
20	-0.2263	-0.2182	-0.2204	-0.2216 ± 0.0042
25	-0.2642	-0.2659	-0.2749	-0.2683 ± 0.0058

Table S4. Establishment of a standard curve for trace amounts of CA using two points method

enzyme activity (U/mL)	slope k_1	slope k_2	slope k_3	average slope k
2	0.0560	0.0560	0.0569	0.0562 ± 0.0005
5	0.0804	0.0803	0.0755	0.0787 ± 0.0028
10	0.1188	0.1022	0.1250	0.1153 ± 0.0118
15	0.1656	0.1603	0.1661	0.1640 ± 0.0032
20	0.2092	0.2146	0.1901	0.2046 ± 0.0129
25	0.2646	0.2488	0.2392	0.2508 ± 0.0128

Table S5. Determination of CA activity half-life in rats by conventional standard curves (0-30min)

Time(s)	sample 1 (U/mL)	sample 2 (U/mL)	sample 3 (U/mL)	sample 4 (U/mL)	sample 5 (U/mL)	sample 6 (U/mL)	average results (U/mL)
0	599.81	720.52	718.67	752.14	518.48	787.10	682.78 ± 102.26
2	477.10	634.38	527.62	621.90	423.62	650.76	555.90 ± 93.83
4	387.24	456.38	453.43	502.00	323.52	478.14	433.45 ± 66.09
6	289.62	356.71	317.71	363.29	252.48	347.14	321.16 ± 43.45
8	194.52	268.19	242.76	284.76	193.81	277.62	243.61 ± 40.85
10	149.67	229.33	149.67	229.95	147.10	218.48	187.37 ± 42.44
12	124.57	181.05	133.62	175.38	134.86	180.14	154.94 ± 26.51
14	96.67	152.62	106.67	148.62	111.90	153.00	128.25 ± 25.89
16	73.90	125.00	77.29	110.76	85.43	134.81	101.20 ± 25.90
18	53.76	113.19	64.76	100.24	70.86	124.67	87.91 ± 28.76
20	36.52	91.05	52.24	95.52	69.43	110.29	75.84 ± 28.10
25	23.98	66.33	34.50	62.10	62.81	76.43	54.36 ± 20.39
30	20.25	53.48	25.52	42.57	37.05	49.76	38.10 ± 13.19

Table S6. Determination of CA activity half-life in rats by trace standard curve (30-60min)

Time(s)	sample 1 (U/mL)	sample 2 (U/mL)	sample 3 (U/mL)	sample 4 (U/mL)	sample 5 (U/mL)	sample 6 (U/mL)	average results (U/mL)
45	11.12	31.02	22.20	28.27	23.68	25.53	23.64 ± 6.90
60	2.77	18.60	14.67	20.03	17.88	4.43	13.06 ± 7.56
